# Profiling vitamin D, its mediators and proinflammatory cytokines in rheumatoid arthritis: A case–control study

**DOI:** 10.1002/iid3.676

**Published:** 2022-07-12

**Authors:** Samuel A. Sakyi, Mavis Owusu‐Yeboah, Christian Obirikorang, Richard K. Dadzie Ephraim, Alexander Kwarteng, Stephen Opoku, Bright O. Afranie, Ebenezer Senu, Andy O. Boateng, Derrick K. Boakye, Tonnies A. Buckman, Benjamin Amoani

**Affiliations:** ^1^ Department of Molecular Medicine, School of Medicine and Dentistry Kwame Nkrumah University of Science and Technology Kumasi Ghana; ^2^ Department of Medical Laboratory Sciences, Faculty of Allied Health University of Cape Coast Cape Coast Ghana; ^3^ Department of Biochemistry and Biotechnology Kwame Nkrumah University of Science and Technology Kumasi Ghana; ^4^ Department of Medical Diagnostics, Faculty of Allied Health Sciences, College of Health Sciences Kwame Nkrumah University of Science and Technology Kumasi Ghana; ^5^ Department of Biomedical Science, School of Allied Health Sciences University of Cape Coast Cape Coast Ghana

**Keywords:** mediators of vitamin D, parathyroid hormone, phosphorus, proinflammatory cytokines, rheumatoid arthritis

## Abstract

**Introduction:**

The active form of vitamin D has immunomodulatory and anti‐inflammatory effect. Vitamin D is implicated in pathogenesis of rheumatoid arthritis (RA) and its deficiency leads to increased inflammation. Moreover, its production is dependent on concentration of calcium, phosphorus, and parathyroid hormone (PTH). Cytokines mediates inflammation in RA synovium. This study evaluated vitamin D, its mediators and proinflammatory cytokines among RA patients.

**Methods:**

In a case–control study, 78 RA patients from Komfo Anokye Teaching Hospital rheumatology clinic and 60 healthy blood donors were recruited. Chemistry analyzer and enzyme‐linked immunosorbent assay kits were used to measure biochemical parameters and cytokines.

**Results:**

We found significantly higher levels of interleukin (IL)‐1β, interferon gamma (IFN‐γ), and tumor necrosis factor‐α (TNF‐α) in RA patients compared with controls (*p* < .05). There was a significant positive correlation between intact parathyroid hormone (iPTH) and IL‐10 (*r* = .30, *p* < .05) and a negative correlation between IL‐6 (*r* = −0.28, *p* > .05), IL‐1β (*r* = −0.25, *p* > .05), TNF‐α (*r* = −0.26, *p* > .05), IFN‐γ (*r* = −0.24, *p* > .05), and iPTH. There was a significant negative correlation between IL‐1β (*r* = −0.33, *p* < .05), IFN‐ γ (*r* = −0.29, *p* < .05), and calcium.

**Conclusion:**

Reduced PTH, calcium, and phosphorus is associated with higher levels of proinflammatory cytokines which may worsen RA disease condition. Vitamin D is therefore not an independent regulator of proinflammatory cytokines in RA.

## Graphical abstract

The primary role of vitamin D has been established in mineral homeostasis, however, its production is dependent on serum concentration of calcium, phosphorus and parathyroid hormone. We found significantly higher levels of proinflammatory cytokines in RA patients compared with the healthy controls. A negative correlation was found between the proinflammatory cytokines (IL‐6, INF‐γ, IL‐1β, and TNF‐α) and the mediators of vitamin D (Ca, iPTH, and phosphorus); however, the anti‐inflammatory cytokine, IL‐10 correlated positively with the mediators of vitamin D.

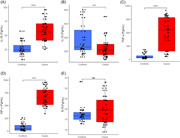

## 1 INTRODUCTION

Rheumatoid arthritis (RA), is a chronic, autoimmune and inflammatory disease, whose cause remains unclear. RA is primarily associated with the synovial joints, with prevalence ranging between 0.5% and 1.0% in the industrialized world.^1^, ^2^ The burden of RA in Africa is presently uncertain, as very few epidemiological studies have been conducted.^3^ An estimated prevalence of 0.2%–0.3% has been reported in Africa.^4^ The primary role of vitamin D has been established in mineral homeostasis; however, its production is dependent on serum concentration of calcium, phosphorus, and parathyroid hormone (PTH).^5^ The active form of vitamin D is believed to have immunomodulatory and anti‐inflammatory effect, with therapeutic potential.^6^ Vitamin D is implicated in RA pathogenesis and its deficiency has been linked to increased joint inflammation. Cytokines mediate inflammation in RA synovium with accompanied joint destruction.^7^ Despite the significant role of both vitamin D and proinflammatory cytokines in the pathogenesis of RA, the interplay between these two in RA is still unclear.

Cytokines are expressed in the synovial tissues where they are functionally active.^8^ Proinflammatory cytokines enhance autoimmune inflammation and tissue damage.^9^ The pathogenesis of RA is known to be promoted by proinflammatory cytokines including tumor necrosis factor‐α (TNF‐α), interleukin‐1β (IL‐1β), IL‐6, IL‐17, and interferon‐γ (IFN‐γ). The synergestic effect of these cytokines is manifested in the increase rate of leukocyte recruitment to the joints, where, they maintain chronic inflammation.^8^ TNF‐α promotes the demineralization of bones by activating osteoclasts, induce proliferation of fibroblast‐like synoviocytes (FLS), pannus formation, and cartilage damage.^10^ IFN‐γ promotes the expression of TNF‐α and IL‐1, with the latter two inflammatory cytokines, playing a significant role in the pathogenesis and etiology of RA.^11^ In RA, elevated levels of these cytokines are associated with poor outcomes and high disease activity scores (DAS).^12^ We have previously established that Th1‐ and Th17‐related cytokines predominate in the pathophysiology of RA, with IL‐6 and IL‐17 being principally and differentially expressed based on the severity of the disease.^13^

Vitamin D remains an essential mineral in bone development, with its role as a modulator of the immune system increasingly becoming obvious.^14^ Vitamin D at the cellular level alters the immune response at the onset of infection and inflammation by reducing the level of proinflammatory cytokines produced by the macrophages and T cells. This is achieved by exerting anti‐inflamatory effects on Vitamin D receptor cells including monocytes, macrophages, and T lymphocytes.^15^, ^16^ Recent studies has described vitamin D as anti‐inflammatory mineral with a beneficial role in RA.^15^, ^17^, ^18^

Mediators of vitamin D such as intact parathyroid hormone (iPTH), calcium, and phosphorus have shown to be implicated several other inflammatory conditions such diabetes and atherosclerosis.^19^, ^20^ The parathyroid gland and the kidney tubules express calcium‐sensing receptor (CaSR) that regulate calcium homeostasis. CaSR regulates by modulating the synthesis and release of PTH and active vitamin D, which enhance calcium transition across the bone, intestine, and kidney. In addition, CaSR play a key role in modulating the immune system by acting as a responder to inflammatory cytokines.^20^ In acute tissue injury, there is an elevation of extracellular calcium that serve as a chemoattractant of macrophages and monocytes to these sites.^21^ Elevated levels of extracellular calcium also mediate the levels of the proinflammatory cytokines.^22^ IL‐1 and IL‐6 activation of parathyroid and renal CaSR results in hypocalcemia, hypovitaminosis D, and hypoparathyroidism. In an animal model, intraperitoneal injection of IL‐6 resulted in 24h decrease in plasma levels of PTH, vitamin D, and calcium.^23^ Critically ill sepsis and burns patients with elevated proinflammatory cytokine levels commonly have hypocalcemia.^24^, ^25^, ^26^

Inorganic phosphorus has also been implicated in the regulation of inflammatory cytokines, although the exact mechanism is not clearly understood. Among sepsis patients, elevated levels of inflammatory cytokines were found to be associated with hypophosphatemia.^27^ In chronic kidney disease patients, phosphorus correlated negatively with inflammatory cytokines, indicating the role of phosphorus in inflammation.^28^ Studies evaluating the interplay between vitamin D mediators (calcium, intact PTHs, and phosphorus) and inflammatory cytokines within hitherto uncommon RA in Ghanaian context has not been explored. Against this background, for the first time, we profiled the association between mediators of vitamin D and inflammatory cytokines in Ghanaian RA patients.

## 2 MATERIALS AND METHODS

### 2.1 Study design and study site

This case–control study was conducted at the rheumatology clinic of the Komfo Anokye Teaching Hospital (KATH) in the Ashanti Region, Ghana. KATH is lies at 6° 41' 50.92'' N and 1° 37' 54.08'' W in the Kumasi Metropolitan Assembly, which has a population size of 1,730,249 (Ghana Statistical Service, 2010). KATH is the major teaching hospital in the middle belt of Ghana with more than 1000 bed capacity and serves as a referral center for other hospitals within and outside the Ashanti Region. Previous study shows high prevalence of 25‐hydroxyvitamin D deficiency among the general adult population in Ghana despite the abundance of sunlight maybe due to dietary deficiencies and other related considerations.^13^

### 2.2 Sample size calculation

The sample size was calculated from the formula:

*n* = $\frac{Z^{2}\;\mathrm{PQ}}{e^{2}}$, where: *n* is the required sample size, *P* is the prevalence of RA estimated in Africa = 0.029%,^29^

*Q* = 1 − *P*, *Z* = *z* value at 95% confidence (1.96), and *e* is the margin of error (0.05),

*n* (minimum number of participants) = $\frac{1.96^{2}(0.029)\left(1-0.029\right)}{0.05^{2}}$ = 43.3 (43).

Hence a minimum of 43 participants was required for the study. To increase statistical power, 78 RA patients 18 years and above, and 60 apparent healthy blood donors age and sex‐matched were recruited for the study.

### 2.3 Participant selection

We purposively recruited 78 patients 18 years and above, diagnosed with RA according the American College of Rheumatology/European League Against Rheumatism (ACR/EULAR) 2010, criteria as cases.^19^ Sixty (60) apparent healthy blood donors age and sex‐matched, with no chronic pain, cardiovascular complaints, chronic inflammatory diseases, malaria, tuberculosis, or parasitic infection and who gave informed consent were recruited and used as control. All patients were prednisolone‐naive. Pregnant women, lactating mothers, individuals undergoing hemodialysis or peritoneal dialysis, cancer, HIV patients, liver disease, kidney diseases, or other immunosuppressed patients were excluded.

### 2.4 Ethical consideration

Ethical approval for this study was obtained from the Committee on Human Research, Publication and Ethics (CHRPE) of the School of Medicine and Dentistry, Kwame Nkrumah University of Science and Technology (CHRPE/AP/003/16), and the Research and Development Unit of KATH. Written informed consent was obtained from all participants after the aims and objectives of the study had been explained to them. Participation was voluntary, and respondents were assured about the confidentiality of their data and were at liberty to opt‐out from the study at any time.

### 2.5 Sample collection and processing

Five (5) milliliters of venous blood was drawn from the ante cubital vein, dispensed into well‐labeled gel separator tube and allowed to stand for at least 30 min but not more than 1 h before centrifugation. Centrifugation was conducted at 3000 rpm for 10 min. The sera were carefully aliquoted into well‐labeled cryo‐tubes and stored at −70°C (Thermo Scientific™ Revco™ UxF −Ultra‐Low Temperature Freezers) until ready for use.

### 2.6 Biochemical assay

Auto‐Creatinine (enzyme) Liguicolor reagent was used to estimate serum creatinine concentration photometrically using an automated chemistry analyzer (Human Star 200, scientific analyzer). Calcium ARS III, phosphorus, albumin, and total protein (code number; LC01, LP01, LA03, and LT02) were used to measure ionized calcium, phosphorus, albumin, and total protein, respectively based on spectrophotometric principles using a chemistry auto‐analyzer (LE Scientific Horizon 850). All reagents and samples were brought to room temperature (18°C–25°C) before use.

### 2.7 Vitamin D and vitamin D binding protein assay procedure

Reagents for the Vitamin D assay were obtained from Biobase Biodustry Co. Ltd. (Lot# 201903). Serum Vitamin D [25(OH)D3] was measured using the Sandwich Enzyme‐Linked Immunosorbent Assay (ELISA) method. We followed manufacturer's protocol for all other procedures. Reagents for the Vitamin D binding protein assay were obtained from Biobase Biodustry Co. Ltd. (Lot# 201903). Serum Vitamin D [25(OH)D3] was measured using the Sandwich ELISA method. We followed manufacturer's protocol for all other procedures. According to information supplied by the manufacturer, 25‐OH Vitamin D ELISA assay kit uses a newly designed monoclonal antibody which is specific for both vitamin D2 and vitamin D3 at 100% specificity.

### 2.8 Cytokine assays

Cytokines measurements were done using the commercially available assay (Human IL‐1, TNF‐α, IL‐10, and IFN‐γ Duoset ELISA Kit principle) from the RD Systems. The manufacturer's protocol was strictly adhered to. The commercially available assay (Human IL‐6 ELISA Kit principle) was obtained from Biobase Biodustry Co. Ltd. The manufacturer's protocol was strictly adhered to. According to information supplied by the manufacturer, the intraassay CVs were 4.9% at a 25(OH)D mean concentration of 27.0 nmol/L, 6.9% at a 25(OH)D mean concentration of 61.5 nmol/L and 3.2% at a 25(OH)D mean concentration of 160.3 nmol/L, respectively.

### 2.9 iPTH assay procedure

All reagents were purchased from Biobase Biodustry Co. Ltd. (Lot# 201903). Serum iPTH was measured using the Sandwich ELISA method. The manufacturer's protocol was strictly adhered to.

### 2.10 Statistical analysis

Data were entered and managed using Microsoft Excel 2016. All analyses were done with the R statistical computing version 4.0.2.^30^ Parametric data were represented as mean ± standard deviation (SD) whilst nonparametric data were presented as median (IQR). Data distribution was assessed using Shapiro–Wilk test and depending on the result, either Student's *t* test or Mann–Whitney test was used to assess the difference between the two groups. Correlation analysis of cytokines with Vitamin D levels and of cytokines with biochemical parameters were done using Spearman's rank correlation test. *p* ≤ .05 was considered statistically significant.

## 3 RESULTS

This case–control study consisted of 78 RA patients and 60 matched healthy controls. Baseline characteristics of the study population are shown in Table 1. The ages of the cases and controls were similar (47.50 ± 15.61 vs. 42.65 ± 7.69, *p* = .175). The distribution of gender was uniform among cases and controls (*p* > .05). There was no statistically significant difference in the serum levels of vitamin D, creatinine, calcium, albumin, and total protein between RA patients and controls (*p* > .05). We observed significantly lower levels of serum inorganic phosphorus and iPTH in RA patients relative to controls (*p* < .05).

**Table 1:** Baseline characteristics of study participants

Variable	Controls (*n* = 60)	RA patients (*n* = 78)	*p* value
Sex (female/male)	36/24	51/27	.516
Age (years)	42.65 ± 7.69	47.50 ± 15.61	.175
25VD (pg/ml)	17.86 ± 3.56	17.48 ± 4.84	.715
DBP (pg/ml)	93.54 ± 18.21	104.67 ± 29.97	.152
iPTH (pg/ml)	49.67 (5.22)	44.16 (11.62)	**.018**
Creatinine (µmol/L)	102.33 ± 57.66	106.94 ± 39.28	.359
Inorganic phosphorus (mmol/L)	1.35 ± 0.35	1.00 ± 0.21	**<.001**
Calcium (mmol/L)	1.87 ± 0.21	1.80 ± 0.23	.326
Albumin (mg/dL)	42.95 ± 4.51	42.64 ± 4.75	.825
Total protein (g/L)	72.10 ± 5.88	72.60 ± 6.30	.863

*Note*: Parametric data were represented as mean ± SD whilst nonparametric data were presented as median (IQR), Student's *t* test (parametric), or Mann–Whitney test (nonparametric) was used to assess the difference between the two groups, bolded values; statistically significant.

Abbreviations: 25VD, 25‐hydroxyvitamin D; DBP‐vitamin D binding protein; iPTH, intact parathyroid hormone; IQR, interquartile range; RA, rheumatoid arthritis; SD, standard deviation.

**Figure 1:**
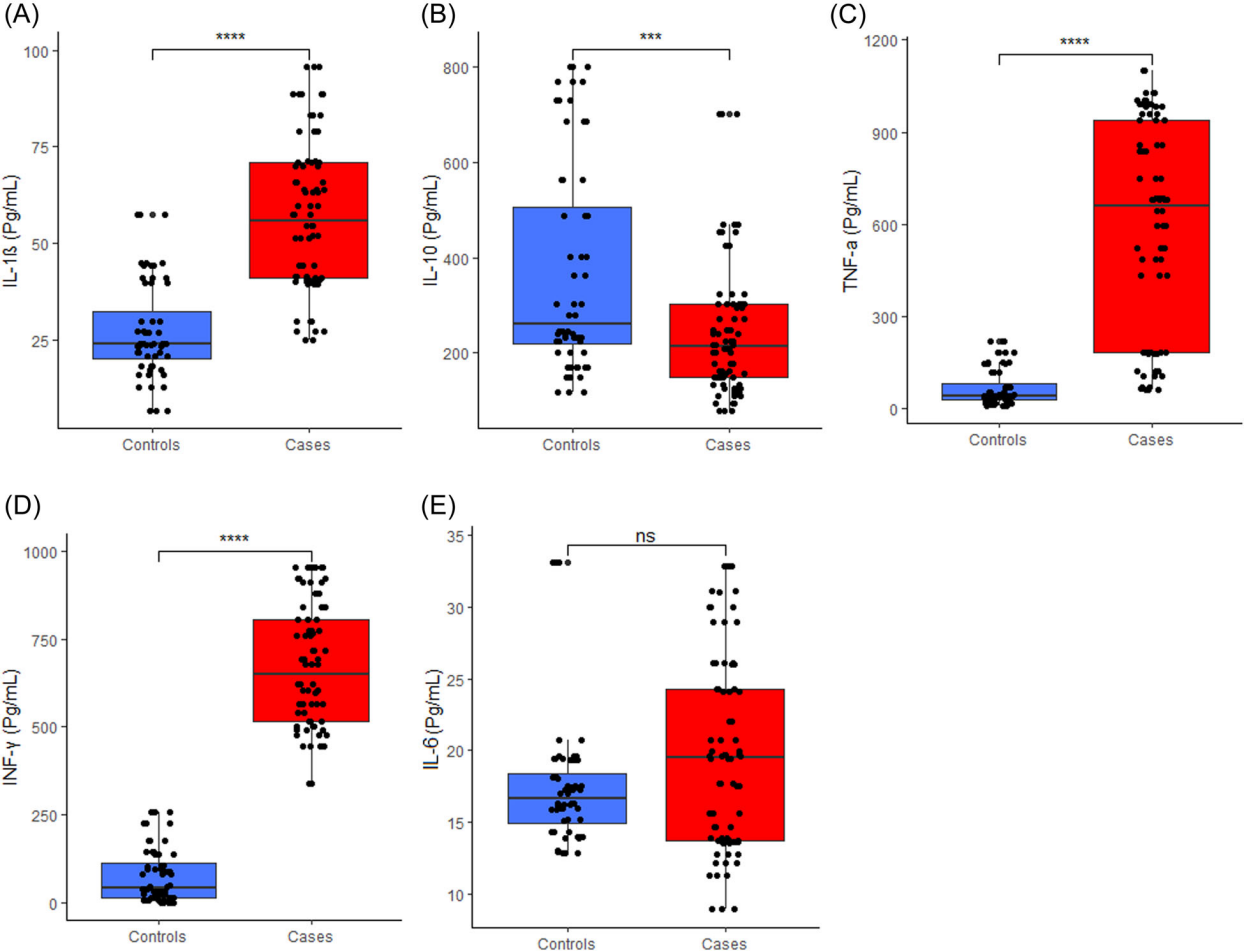
Levels of inflammatory cytokine between controls and RA patients. (A) IL‐1β, (B) IL‐10 (C) TNF‐α, (D) IFN‐γ, and (E) IL‐6. (ns: *p* > .05, *p ≤ .05, **p ≤ .01, ***p ≤ .001, ****p ≤ .0001). IFN‐γ, interferon gamma; IL, interleukin; RA, rheumatoid arthritis; TNF‐α, tumor necrosis factor‐α

Our results also showed significantly higher serum levels of IL‐1β, IFN‐γ, and TNF‐α in RA patients compared with controls (*p* < .05). Conversely, levels of IL‐10 were significantly higher in controls compared to RA patients (*p* < .05). However, we observed no significant difference in serum levels of IL‐6 between RA patients and the healthy controls (Figure 1).

**Figure 2:**
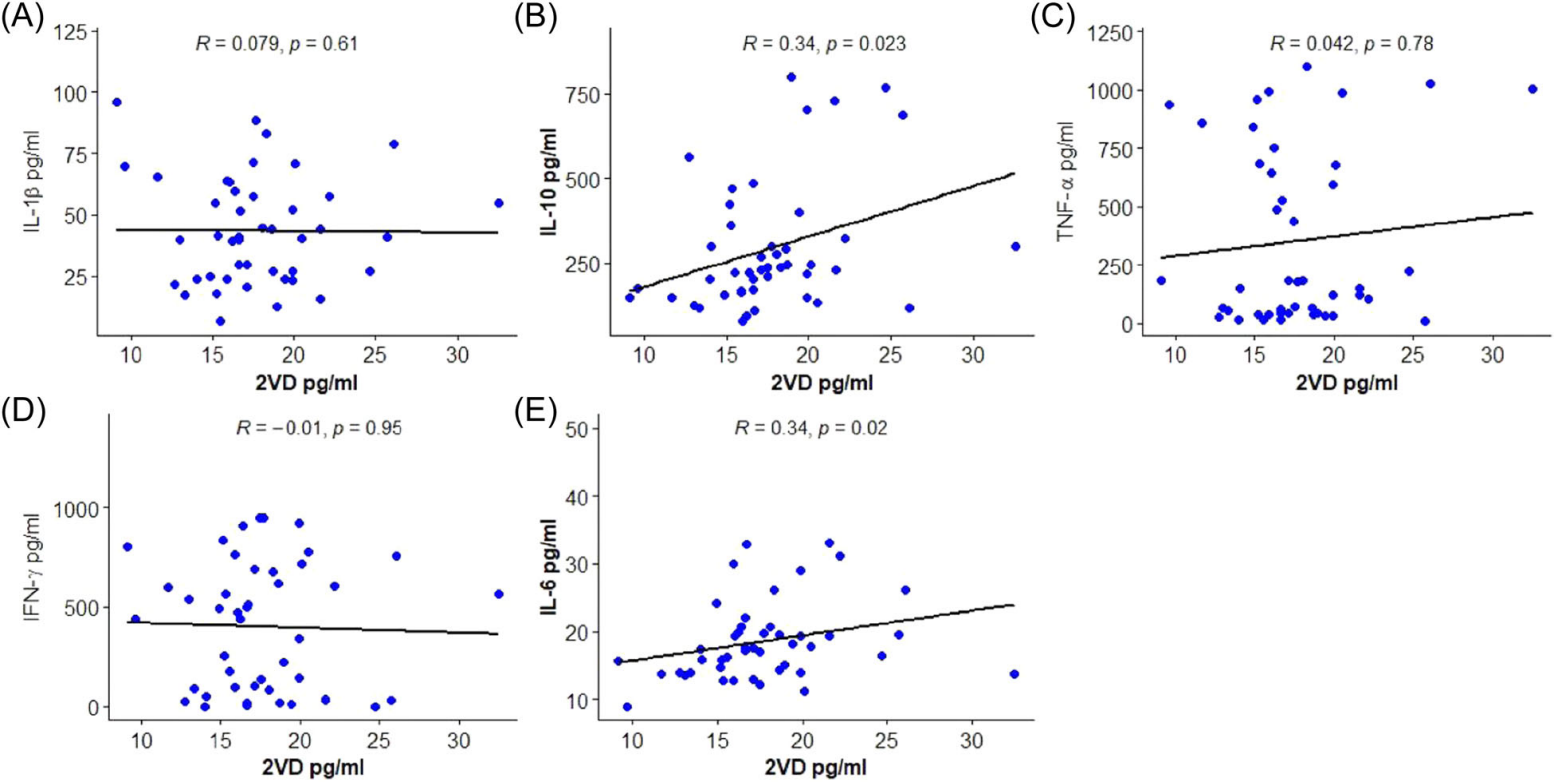
Correlation between 25 VD and cytokines among RA patients: (A) IL‐1β, (B) IL‐10, (C) TNF‐α, (D) IFN‐γ, and (E) IL‐6. IFN‐γ, interferon gamma; IL, interleukin; RA, rheumatoid arthritis; TNF‐α, tumor necrosis factor‐α

Results for the associations between vitamin D and cytokines among RA patients are shown in Figure 2. There were significant positive associations between serum levels of Vitamin D and levels of IL‐6 (*r* = .34, *p* = .020) and IL‐10 (*r* = .34, *p* = .023). Serum levels of IL‐1β, TNF‐α, and IFN‐γ demonstrated no statistically significant association with serum vitamin D levels (*p* > .05).

Table 2 displays correlation analysis results between cytokines and the mediators of vitamin D among RA patients. There was a statistically significant positive correlation between serum levels of IPTH and IL‐10 (*r* = .30, *p* < .05). We, however, observed a negative correlation between IL‐6 (*r* = −.28, *p* > .05), IL‐1β (*r* = −.25, *p* > .05), TNF‐α (*r* = −.26, *p* > .05), IFN‐γ (*r* = −.24, *p* > .05) and iPTH.

**Table 2:** Correlation between proinflammatory cytokines and mediators of vitamin D among RA patients

Cytokines	iPTH	DBP	ALB	Ca	Cr	TP	Phosphorus
IL‐1β (pg/ml)	−.25	.21	.09	−.33*	−.09	−.02	−.49***
IL‐10 (pg/ml)	.30*	.043	.08	.07	−.42**	.06	.07
TNF‐α (pg/ml)	−.26	.15	.15	−.23	−.11	.07	−.46***
IFN‐γ (pg/ml)	−.24	.31*	.13	−.29*	.15	.13	−.50***
IL‐6 (pg/ml)	−.28	.27	.05	.16	−.03	−.11	−.07

*Note*: Correlation analysis of cytokines with Vitamin D levels and of cytokines with biochemical parameters was done using Spearman's rank correlation test.

Abbreviations: ALB, albumin; Ca, calcium; DBP, vitamin D binding protein; IFN‐γ, interferon gamma; IL, interleukin; iPTH, intact parathyroid hormone; TNF‐α, tumor necrosis factor‐α; RA, rheumatoid arthritis; TP, total protein.

* *p* ≤ .05.

** *p* ≤ .01.

*** *p* ≤ .001.

There was a significant negative correlation between Ca and IL‐1β (*r* = −.33, *p* < .05) and IFN‐γ (*r* = −.29, *p* < .05), a negative but not significant correlation between Ca and TNF‐α (*r* = −.23, *p* > .05). On the contrary, IL‐10 (*r* = .07, *p* > .05) and IL‐6 (*r* = .16, *p* > .05) correlated positively with Ca. Except for IL‐10 (*r* = .07, *p* > 0.05) which correlated positively with Phosphorus, IL1β (*r* = −.49, *p* < .05), TNF‐α (*r* = −.46, *p* < .05) and IFN‐γ (*r* = −.50, *p* < .05) correlated negatively and significantly with Ca. Serum DBP levels demonstrated a significant negative correlation with IFN‐γ. There was no significant association between DBP and IL‐6, IL‐10, IL‐1β, and TNF‐α. For albumin, we observed no significant correlation with all the studied cytokines. There were significant negative associations between calcium and IL‐1β and IFN G. For creatinine, there was a significant negative correlation with IL10. There was no significant correlation between creatinine and IL‐6, IL‐1β, TNF‐α, and IFN‐α (*p* > .05). Serum levels of total protein had no significant correlation with IL‐6, IL‐10, IL1β, TNF‐α, and IFN‐α (*p* > .05).

## 4 DISCUSSION

Mediators of vitamin D (calcium, phosphorus, and parathroid hormone) are known to regulate inflammatory cytokines in both animal studies and human diseases. Unfortunately, studies evaluating the interplay between vitamin D mediators and inflammatory cytokines in RA are rare. This current study evaluated the association between mediators of vitamin D and inflammatory cytokines among RA patients. There was no statistically significant difference in the serum levels of vitamin D between RA patients and controls (*p* = .715). We found significantly higher levels of proinflammatory cytokines in RA patients compared to the healthy controls. A negative correlation was found between the proinflammatory cytokines (IL‐6, INF‐γ, IL‐1β, and TNF‐α) and the mediators of vitamin D (Ca, iPTH, and phosphorus). However, the anti‐inflammatory cytokine, IL‐10 correlated positively with the mediators of vitamin D.

The observed finding of no statistically significant difference in the serum levels of Vitamin D between RA patients and controls confirms a previous study by Sakyi et al.,^13^ who found a high prevalence of 25‐hydroxyvitamin D deficiency among the general adult population in Ghana despite the abundance of sunlight. This could be attributed to dietary deficiencies and other related considerations of the general population.^13^

Proinflammatory cytokines including TNF‐α, IL‐1β, IL‐6, and IFN‐γ play a significant role in the pathogenesis of RA. These cytokines act synergetically leading to an increase in the rate of leukocyte recruitment to the joints, where, they maintain chronic inflammation.^8^ TNF‐α promotes the demineralization of bones by activating osteoclasts and subsequent cartilage damage.^10^ Elevated levels of these cytokines have been reported in RA and may be associated with poor outcomes and high disease activity scores (DAS).^12^

In this study, the RA patients had significantly higher levels of the proinflammatory cytokines; IL1β, IFN‐γ, and TNF‐α between RA patients and the healthy controls, except for IL‐6 (Figure 1). TNF‐α has been tagged as the principal cytokine in RA pathogenesis as it regulates the formation of other proinflammatory cytokines.^31^ In a Ghanaian study, intracytoplasmic expression of TNF‐α was higher in RA patients than in healthy controls.^32^ A study by Edress et al.,^33^ found significantly higher TNF‐α levels in synovial fluid and serum of RA patients relative to the healthy controls. These findings are consistent with our current study suggesting that TNF‐α is greatly involved in the pathogenesis of RA. Similarly, IL‐1β has been associated with some proinflammatory activities such as the T cell activation and promotion of proinflammatory cytokines production.^34^ IL‐1β has also been reported to be associated with increased disease activity in RA patients.^35^ Mateeen et al., demonstrated elevated levels of IL‐1β in synovial fluid and serum of RA patients.^12^ The current study also observed high IL‐1β levels among RA subjects.

IFN‐γ is a characteristic Th1 cell proinflammatory cytokine with an unclearly defined role in RA. IFN‐γ has been described to have a protective function via the suppression of osteoclast formation, macrophages activation, and neutrophils recruitment.^36^, ^37^ Sakyi et al.,^32^ observed significantly higher levels of IFN‐γ in RA patients compared to healthy controls. Similarly, Paramalingam et al.,^38^ reported significantly higher levels of IFN‐γ among RA patients relative to controls. Such findings are consistent with this current study suggesting the involvement of IFN‐γ is in the RA pathogenesis.

IL‐6 acts on neutrophils and releases proteolytic enzymes and reactive oxygen intermediates thus promoting inflammation and joint destruction.^12^ Increased levels of IL‐6 have been found in blood and synovial fluids of RA patients, and are reported to promote angiogenesis in synovial fibroblast.^39^ Ding et al. have reported IL‐6 to positively correlate with markers of inflammation in RA patients.^12^ Even though this current study found no statistically significant difference in levels of IL‐6 between RA patients and healthy controls, there seemed to be relatively higher serum levels in RA patients compared with healthy controls (Figure 1).

Interestingly in this study, levels of IL‐10 were significantly higher in controls compared to RA patients. IL‐10, an anti‐inflammatory cytokine, downregulates proinflammatory cytokines production and also functions in the suppression of antigen‐presenting cell functions.^40^ Some studies have shown raised serum levels of IL‐10 in RA patients relative to healthy controls.^41^, ^42^ However, detection of relatively lower serum levels of IL1‐0 in RA patients in this current study may suggest a reduction in anti‐inflammatory activities geared toward the improvement of RA symptoms and hence increased inflammatory damage. This claim is corroborated by studies conducted on animal models of arthritis, which revealed the role of IL‐10 in the reduction of arthritis severity.^43^

Several studies have examined the relationship between serum Vitamin D levels and serum levels of cytokines. Some studies showed a significant positive relationship between serum IFN‐γ and IL‐10,^44^ others showed a significant negative relationship between IL‐6 and IL‐10,^45^ or no significant changes in the serum levels of IL 6, IL‐10, IFN‐γ, and TNF‐α.^46^, ^47^, ^48^ From our results, IL‐6 and IL‐10 showed a statistically significant positive correlation with Vitamin D levels. This observation suggests that relatively lower serum Vitamin D levels in RA patients may not be the sole contributor to the relatively higher serum levels of the studied cytokines in the RA patients.

An important finding in this study is the negative correlation between the proinflammatory cytokines; IL6, INF‐γ, IL‐1β, TNF‐α, and iPTH. Also, serum inorganic phosphorus correlated negatively with IL‐1β, TNF‐α, and IFN‐γ. Furthermore, we demonstrated a negative correlation between IL‐1β, TNF‐α IFN‐γ, and calcium (Table 2). The association between inflammatory cytokines and mediators of vitamin D has not been widely explored in human studies including RA; however, this link has been studied in animal models. In a previous study, injection of IL‐1β and IL‐6 resulted in decreased PTH secretion, depicted by a negative association between IL‐1β, IL‐6, and PTH in an animal model.^49^ In another study, IL‐1β and IL‐6 activation of CaSR in the parathyroid gland and the kidneys resulted in hypocalcemia, hypovitaminosis, and hypoparathyroidism, depicting the interaction between cytokines and mediators of vitamin D.^50^ In chronic myeloid leukemia (CML) patients, Imatinib therapy inhibited IL‐6 levels and a resultant alteration in extracellular calcium.^51^ Inorganic phosphorus has also been implicated in the regulation of inflammatory cytokines in other human diseases. Among sepsis patients, elevated levels of inflammatory cytokines were found to be associated with hypophosphotemia.^27^ In chronic kidney disease patients, phosphorus correlated negatively with inflammatory cytokines, indicating the role of phosphorus in inflammation.^28^

The observed negative correlation between PTH, Ca, phosphorus and inflammatory cytokines confirm that mediators of vitamin D play a role in regulating inflammatory cytokines in RA, ruling out vitamin D as the sole regulator of inflammatory cytokines in RA.

Our study has some limitations, this study was case‐control in design and thus causal inferences cannot be drawn. Furthermore, it was a single‐center study hence findings may not be generalizable. Again, the sample size was modest and could increase in future studies whilst exploring other mediators. Our study also did not collect data on the diet and exercise of study participants since hormones such as vitamin D, PTH, and minerals like calcium are affected by diet and exercise.

## 5 CONCLUSION

Reduced PTH, calcium, and phosphorus are associated with higher levels of proinflammatory cytokines which may worsen RA disease condition. Vitamin D is therefore not an independent regulator of proinflammatory cytokines in RA.

## AUTHOR CONTRIBUTIONS

Samuel Asamoah Sakyi, Mavis Owusu‐Yeboah, Christian Obirikorang, Richard K. Dadzie Ephraim, Alexander Kwarteng, and Benjamin Amoani did the study conceptualization. Samuel Asamoah Sakyi, Christian Obirikorang, Richard K. Dadzie Ephraim, Alexander Kwarteng, and Benjamin Amoani did the study supervision. Samuel Asamoah Sakyi, Mavis Owusu‐Yeboah, Christian Obirikorang, Richard K. Dadzie Ephraim, Alexander Kwarteng, Stephen Opoku, Bright Oppong Afranie, Ebenezer Senu, Andy Opoku Boateng, Derrick Kyei Boakye, Tonnies Abeku Buckman, and Benjamin Amoani were in charge of data curation, investigation, and methodology. Samuel Asamoah Sakyi, Christian Obirikorang, and Mavis Owusu‐Yeboah contributed essential reagents and resources. Samuel Asamoah Sakyi, Mavis Owusu‐Yeboah, Christian Obirikorang, Richard K. Dadzie Ephraim, Alexander Kwarteng, Stephen Opoku, Bright Oppong Afranie, Ebenezer Senu, Andy Opoku Boateng, Derrick Kyei Boakye, Tonnies Abeku Buckman, and Benjamin Amoani were in charge of formal analysis, manuscript writing, review, and editing. All authors have read and approved the final manuscript.

## CONFLICT OF INTEREST

The authors declare no conflict of interest.

## Supporting information

Supporting information.

## Data Availability

The data sets used and/or analyzed during the current study are available from the corresponding author on reasonable request.
